# Ask‐Upmark Kidney Disease: Case Report of a Rare Congenital Malformation

**DOI:** 10.1155/crip/5533202

**Published:** 2026-02-20

**Authors:** Raiane Machado Maia, Guilherme Alves Andrade, Heitor Lino Guimarães, Flávio Ricardo Manzi, Luís Cândido Pinto da Silva, Izabella Lucas de Abreu Lima

**Affiliations:** ^1^ Department of Dentistry, School of Dentistry, Pontifical Catholic University of Minas Gerais, Belo Horizonte, Minas Gerais, Brazil

**Keywords:** abnormalities, chronic, drug-induced, gingival overgrowth, renal insufficiency

## Abstract

Ask‐Upmark kidney disease is a congenital renal malformation characterized by segmental hypoplasia, leading to progressive impairment of renal function and severe hypertension. It is a rare anomaly that presents with various complications. A 14‐year‐old boy diagnosed with Ask‐Upmark kidney was referred for dental care. The patient presented with mouth breathing, unsatisfactory occlusion, and severe drug‐induced gingival hyperplasia, which hindered speech, diet, and oral hygiene. Imaging revealed the presence of all permanent teeth, but with delayed eruption, oblique eruption pathways, and risk of impaction. Management included oral hygiene instructions, prophylaxis, and ulectomy to facilitate tooth eruption. This case highlights the oral manifestations associated with the management of Ask‐Upmark kidney, specifically drug‐induced gingival enlargement and eruption disturbances. It underscores the necessity of multidisciplinary treatment, including dentistry, to improve the quality of life for these patients.

## 1. Introduction

Ask‐Upmark kidney was first reported by Ask‐Upmark in 1929 [1]. It is characterized by segmental renal hypoplasia, which can be unilateral or bilateral and is markedly predominant in females [2]. Despite being described many years ago, it remains a rare condition with limited recent reports in the literature.

Physiologically, the condition has repercussions on the central nervous, cardiovascular, digestive, and excretory systems due to severe hypertension and renal insufficiency [3, 4]. Although systemic complications are well‐documented, there is a significant gap regarding the description of oral manifestations in these patients.

The aim of this case report is to describe the oral manifestations associated with Ask‐Upmark kidney disease and to highlight the importance of interdisciplinary management when treating patients with this condition. To the best of our knowledge, this is the first report focusing specifically on the oral features of this congenital anomaly.

## 2. Case Presentation

A 14‐year‐old boy attended the dental clinic following a medical referral from the pediatric nephrology department. A thorough anamnesis was performed, and the parents provided a medical report confirming the diagnosis of Ask‐Upmark kidney. The patient′s main complaint was severe gingival hyperplasia, which was painless but esthetically and functionally compromising.

The patient′s parents (aged 32 and 37) were nonconsanguineous. Regarding the clinical history, the child was born at 42 weeks of gestation. Prenatal care showed no complications or congenital anomalies. The birth was normal but induced after a 15‐day delay beyond the expected date. At birth, the baby was cyanotic but improved within a few hours. He was discharged 2 days later without any diagnosed systemic problems.

Until age two, the patient had recurrent episodes of fever of unknown origin. Subsequently, he exhibited severe weight loss and epilepsy attacks, followed by two episodes of acute myocardial infarction (AMI) and three cerebrovascular accidents (CVA). He was admitted to a specialized hospital with severe hypertension (BP 180/240 mmHg), resulting from obstruction of the aorta.

At two and a half years of age, his left kidney was functioning normally; however, a hypotrophic right kidney was discovered with moderately depressed function. He was diagnosed with renal segmental hypoplasia (Ask‐Upmark kidney). Currently, the patient has secondary chronic kidney disease with stable renal function (glomerular filtration rate of 147 mL/min) and controlled systemic arterial hypertension. He has partial motor deficit on the left side due to the previous stroke and uses crutches.

Current medications include: enalapril 5 mg, hydrochlorothiazide 25 mg, amlodipine 5 mg, hydralazine 25 mg, valproic acid 7 ml, and vitamin D.

Intraoral clinical examination revealed severe gingival hyperplasia (Figure 1), which the father reported appearing around age seven. Despite the hyperplasia, oral hygiene was satisfactory. The patient presented a mixed dentition with prolonged retention of primary teeth (primary maxillary right lateral incisor, primary maxillary right canine, and primary maxillary left canine). No carious lesions or structural enamel defects were observed. The patient is a mouth breather with unsatisfactory occlusion, atretic maxillary and mandibular arches, anterior open bite, and increased overjet. Furthermore, the clinical crowns of his posterior teeth were short, making it difficult for him to eat.

Figure 1Intraoral photographs showing the teeth present and severe gingival hyperplasia (a) front view, (b) right side with teeth out of occlusion, (c) right side in occlusion, (d) left side in occlusion, (e) occlusal view of the maxilla, and (f) occlusal view of the mandible.

**Figure:**
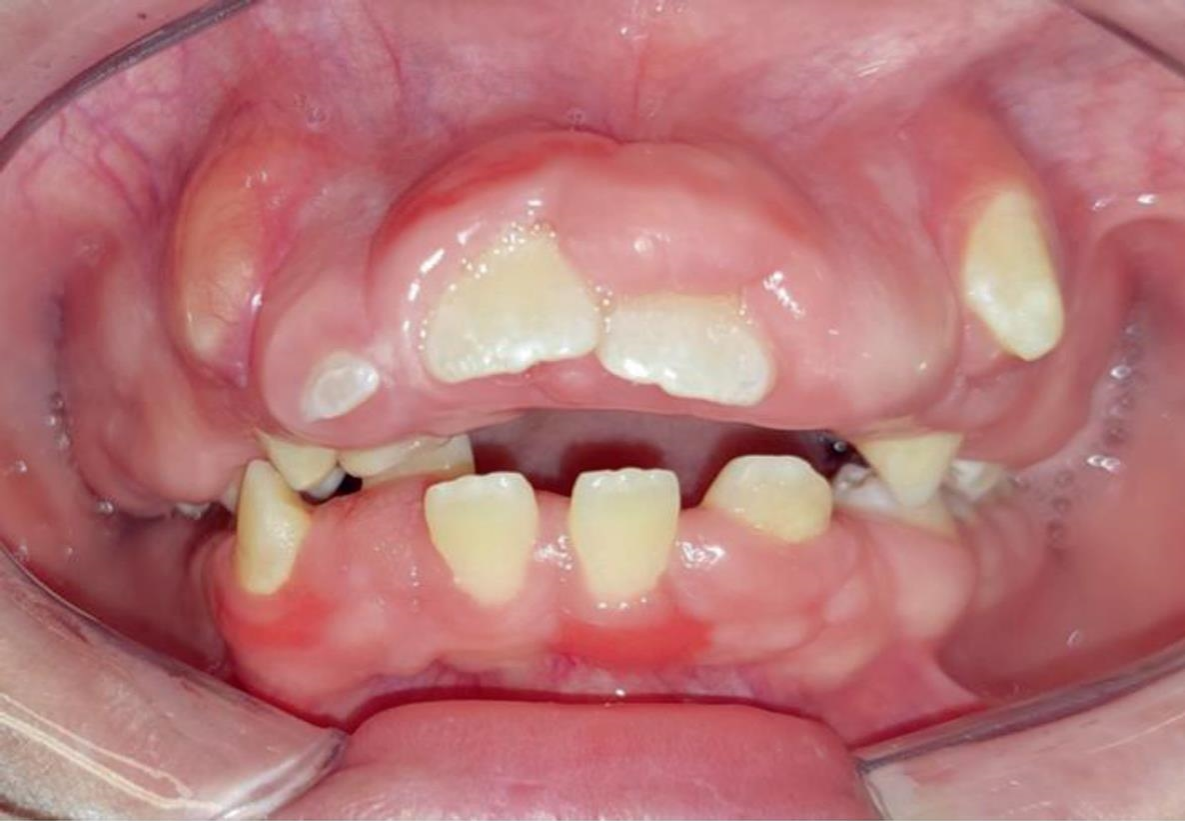
(a)

**Figure:**
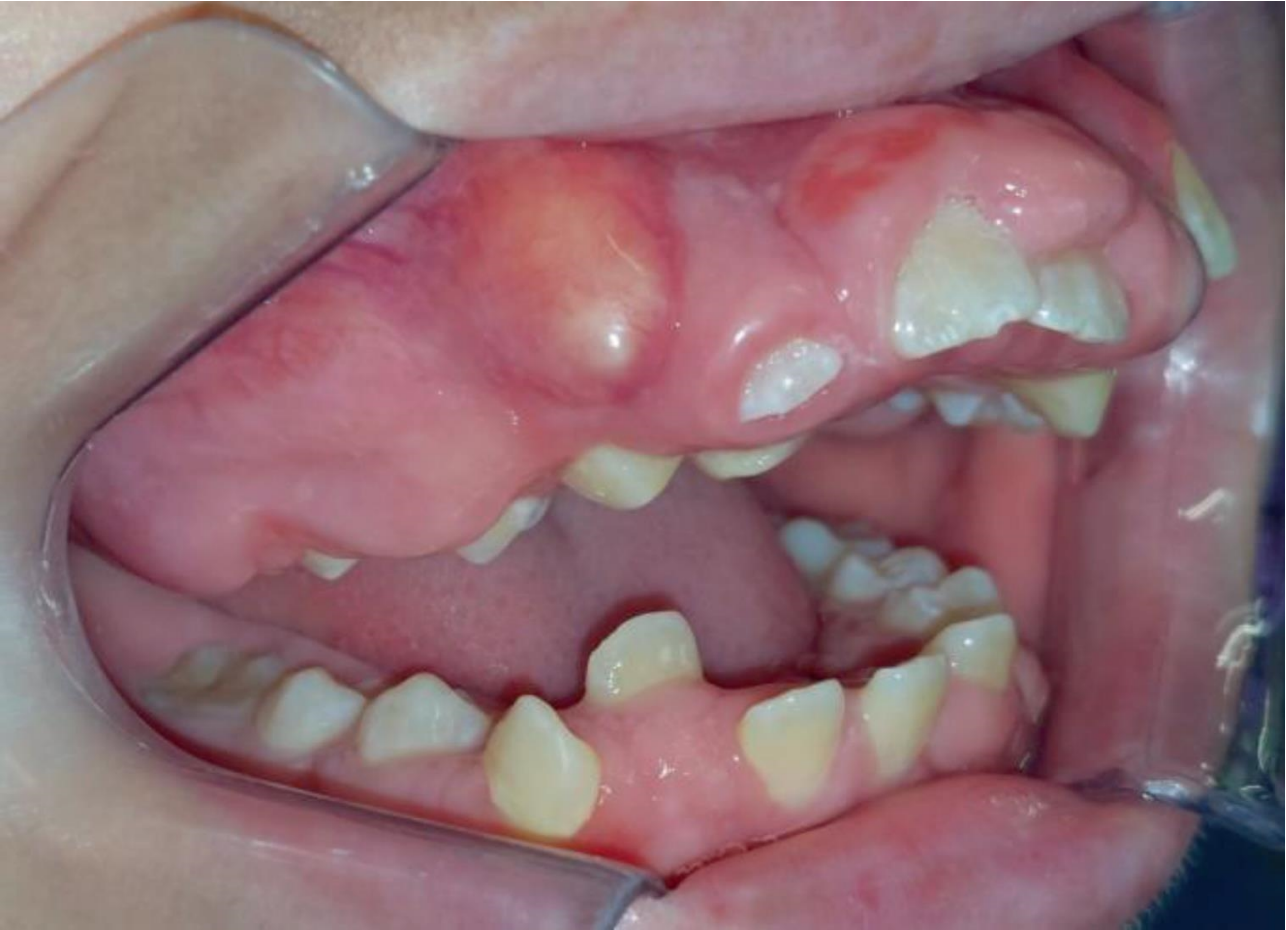
(b)

**Figure:**
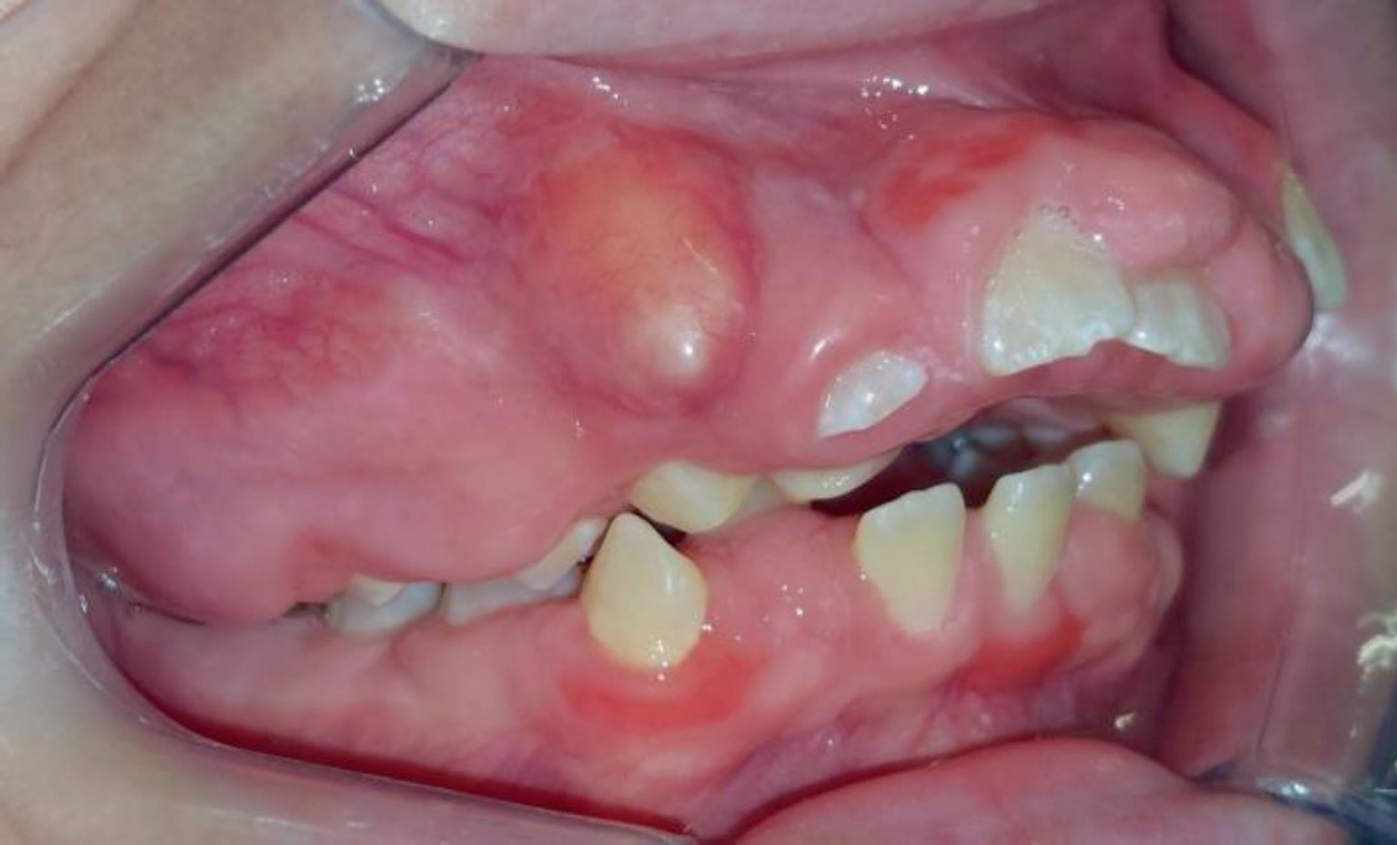
(c)

**Figure:**
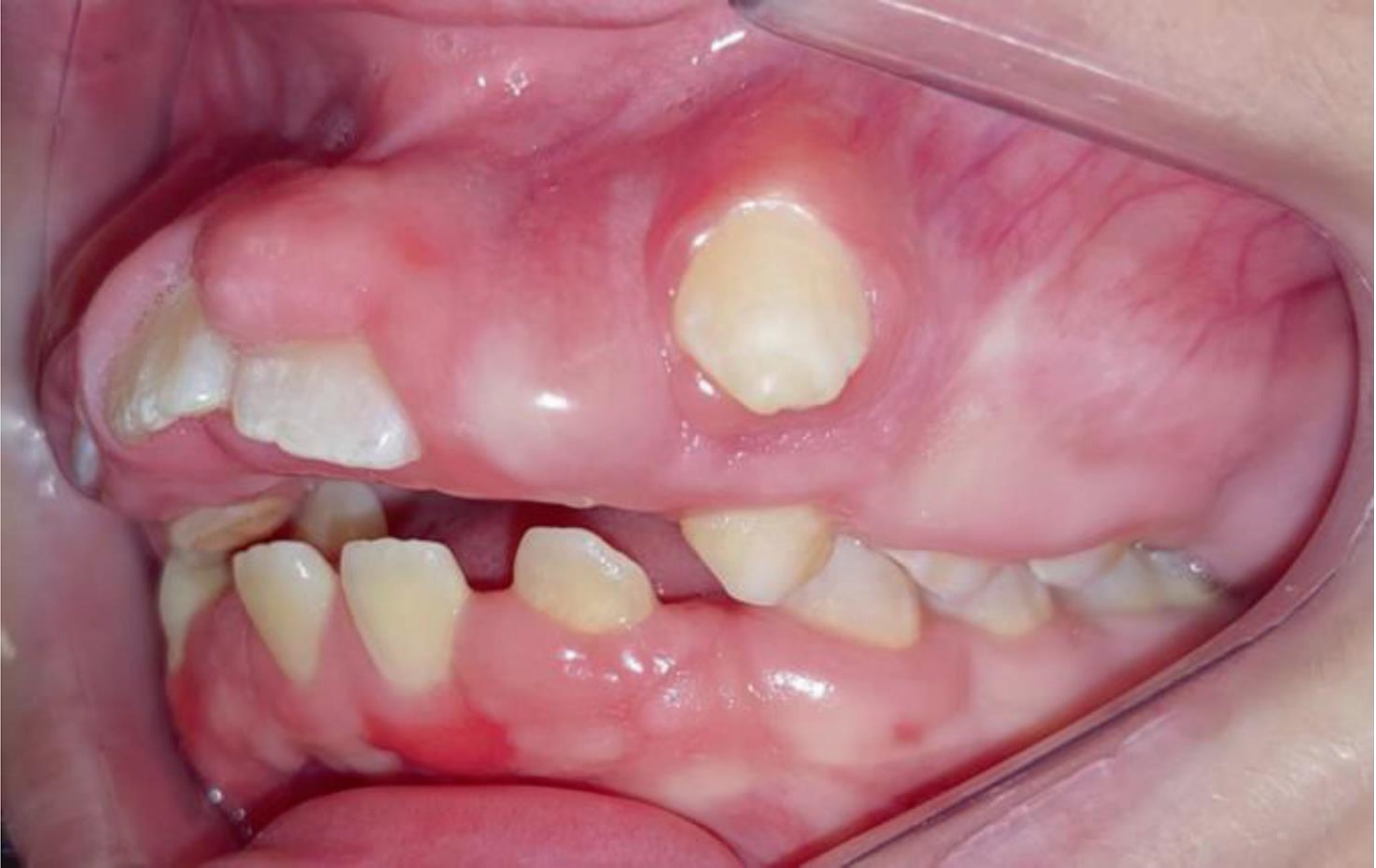
(d)

**Figure:**
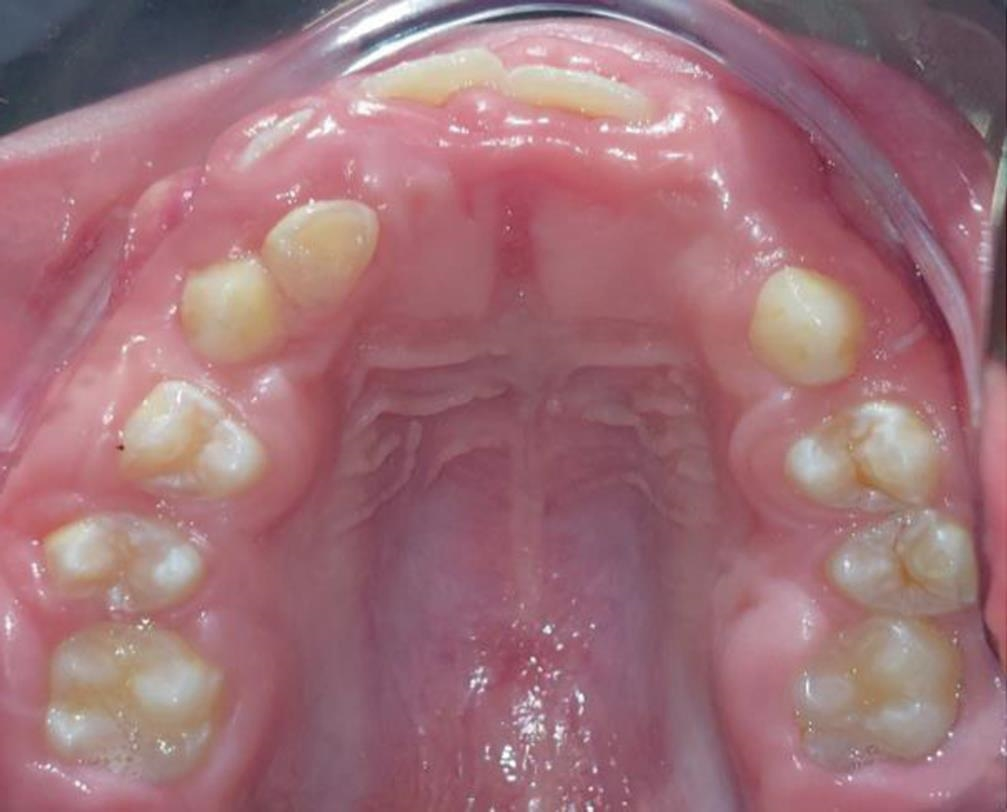
(e)

**Figure:**
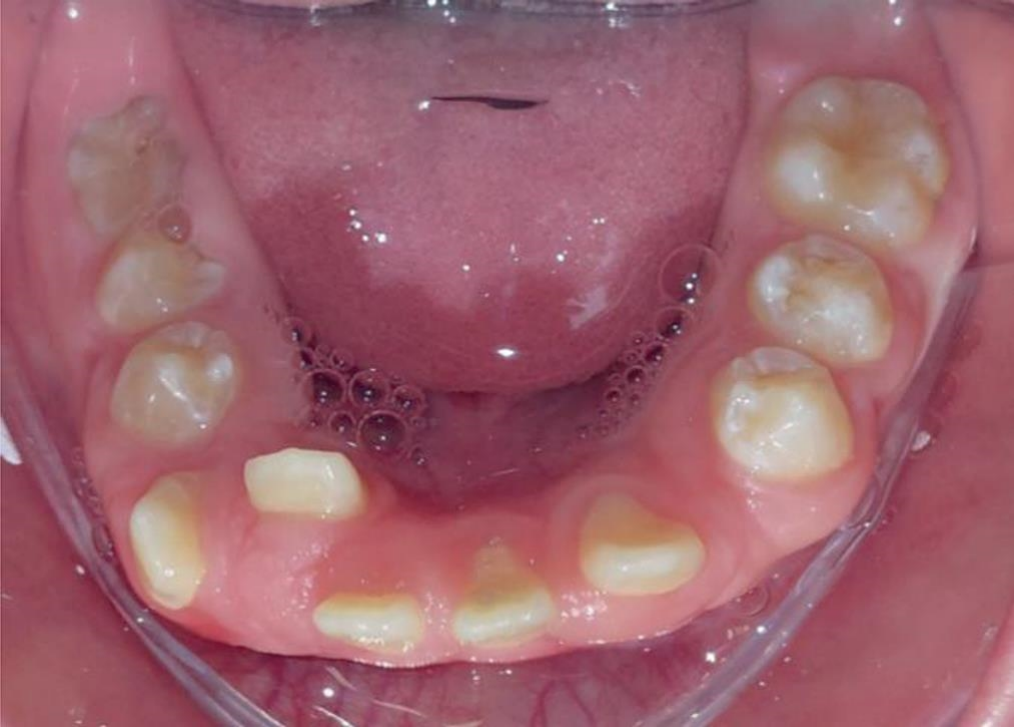
(f)

**Figure 2:**
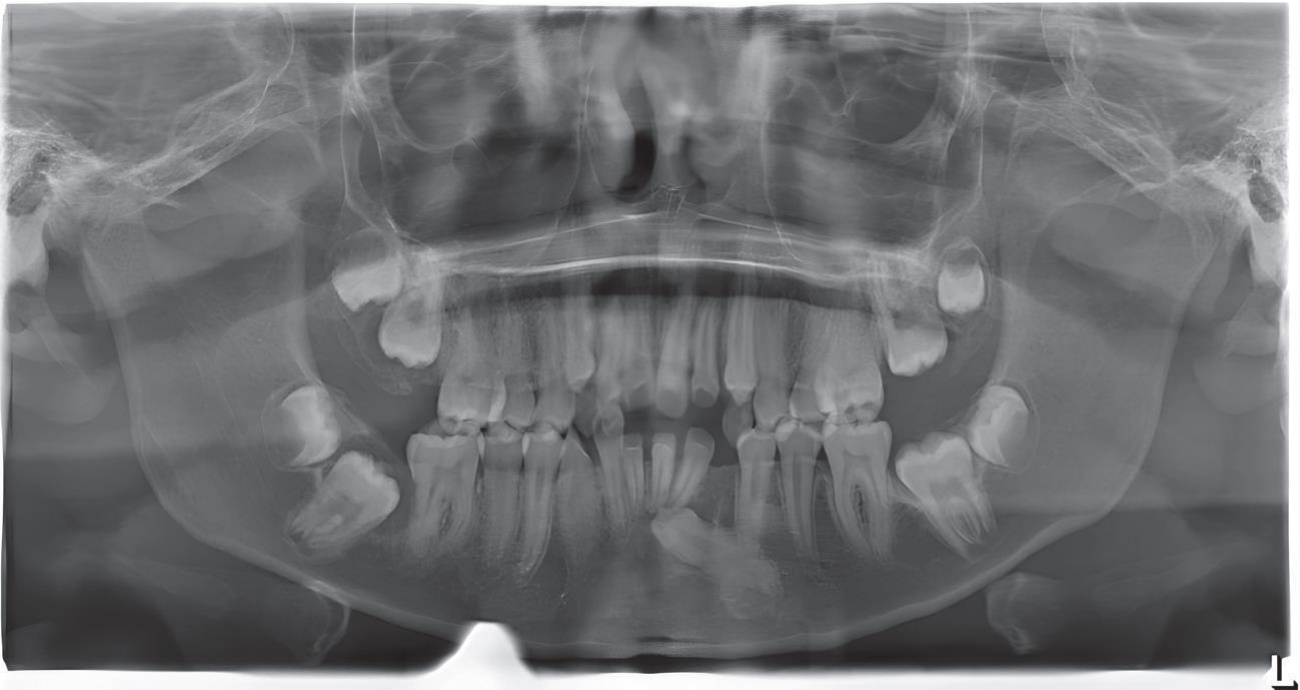
Panoramic radiograph of the 14‐year‐old patient with Ask‐Upmark kidney showing the teeth present. Some teeth show eruption deviation and a great tendency to impaction.

Panoramic radiography (Figure 2) confirmed the presence of all 32 permanent teeth. However, several teeth showed delayed tooth eruption and oblique eruption pathways. Specifically, the permanent mandibular left canine (Tooth 33) was impacted.

**Figure 4:**
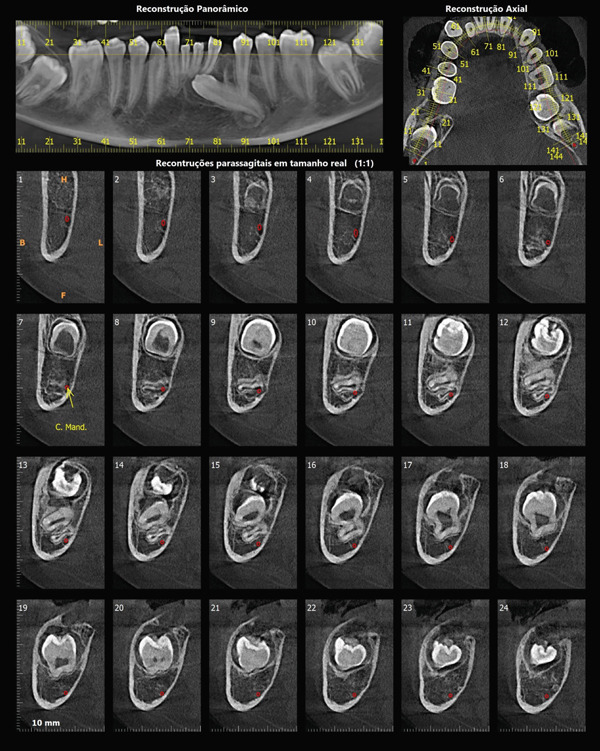
Tomographic images of the mandible. Panoramic, axial, and parasagittal reconstruction. Eruption deviation and impaction of Tooth 33 is observed along with its relationship to adjacent teeth. Teeth 37, 38, 47, and 48 present incomplete rhizogenesis and a great tendency to impaction; they are in close relationship with the mandibular canal, causing luminal stenosis.

High‐resolution cone beam computed tomography (Figures 3 and 4) revealed:

• Teeth in formation: 18, 28, 38, and 48.
• Teeth with incomplete rhizogenesis: 17, 27, 37, and 47, with delayed tooth eruption.
• Tooth 33: impacted in a horizontal position, with the crown facing mesial and buccal, with intimate contact with the roots of Teeth 31 and 32, without promoting root resorption. Notably, the crown perforated the external cortical bone of the mandible, whereas the root remained intraosseous. Presence of pericoronal space suggestive of variation in individual normality or the beginning of the formation of a cystic lesion (dentigerous cyst).
• A hypodense, unilocular image in the left mandibular body suggestive of focal cemento‐osseous dysplasia.
• Hypodense, unilocular, circumscribed, partially corticalized image with heterogeneous hyperdense images in between (varying degree of tomographic density) in the region of the body of the mandible on the left side (inferior to the periapical region of the Tooth 33), with an average diameter of 8.1 mm.
• Diagnosis suggestive of focal cemento‐osseous dysplasia in the cementoblast phase.

Figure 3Tomographic images. Three‐dimensional reconstruction of the maxilla and mandible (a and b) front view, (c) right side view, (d) left side view, and (e and f) view of the mandibular base and overjet.

**Figure:**
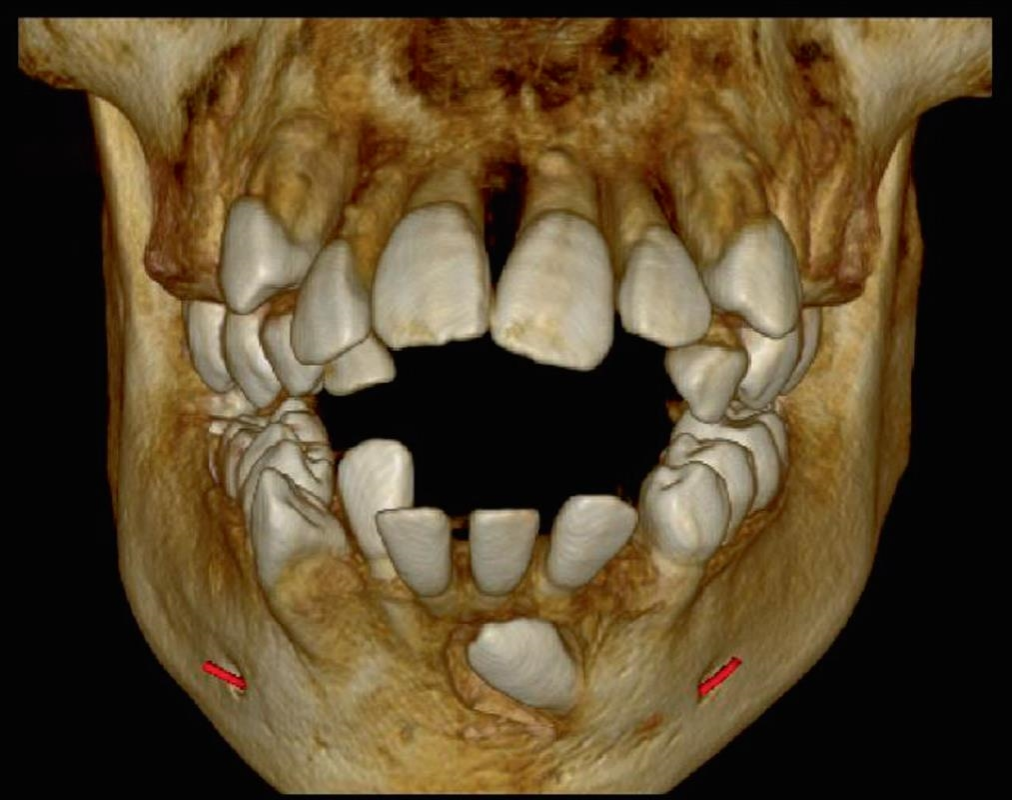
(a)

**Figure:**
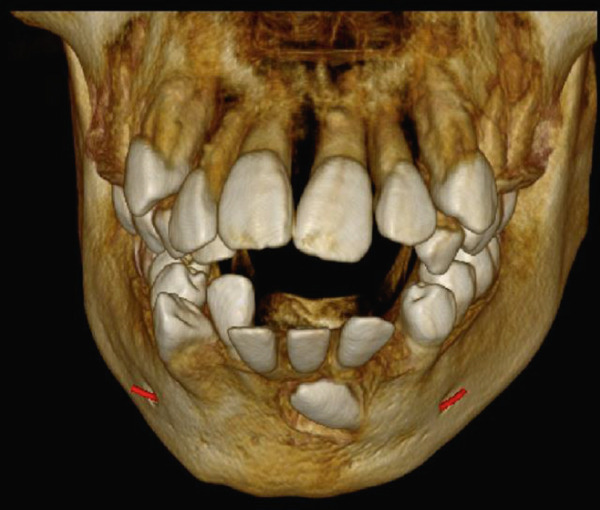
(b)

**Figure:**
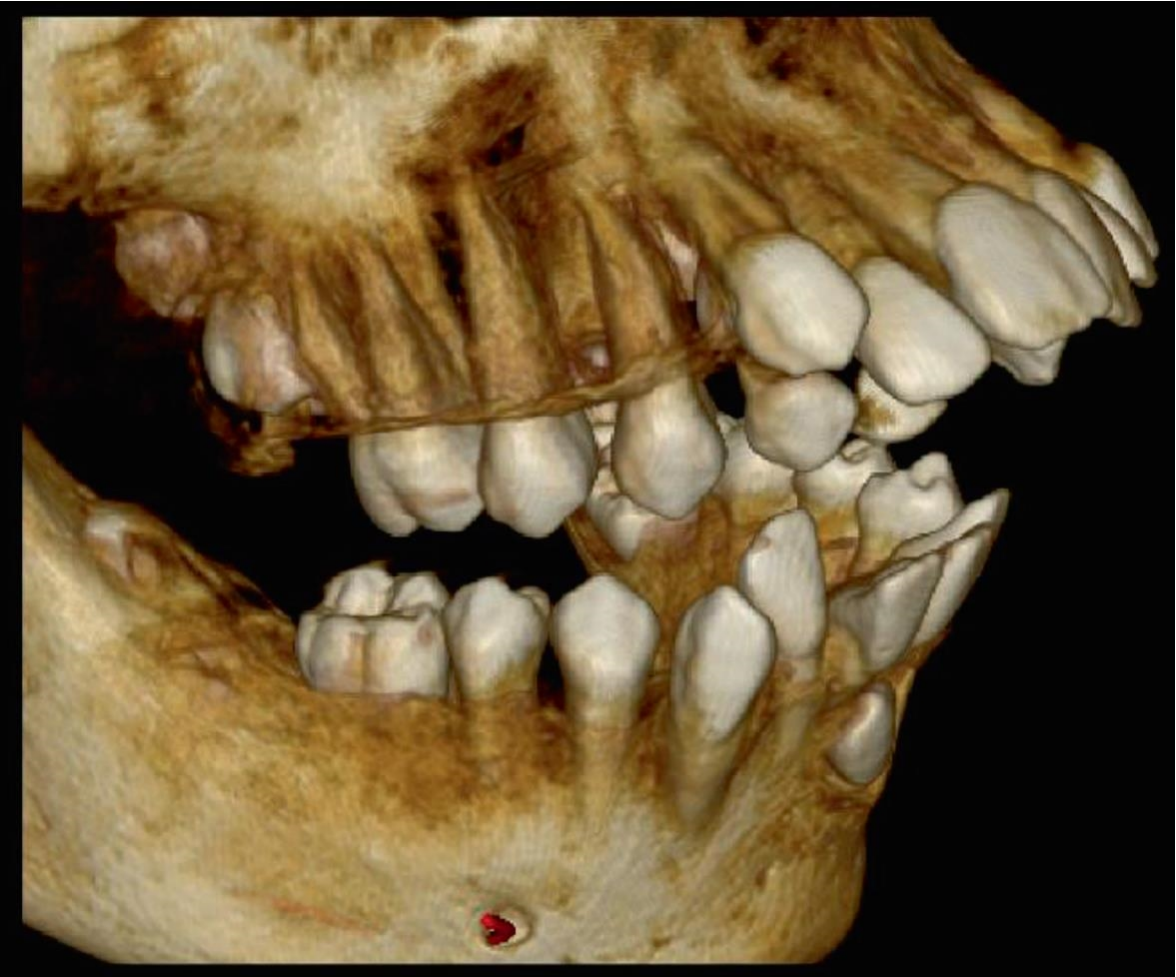
(c)

**Figure:**
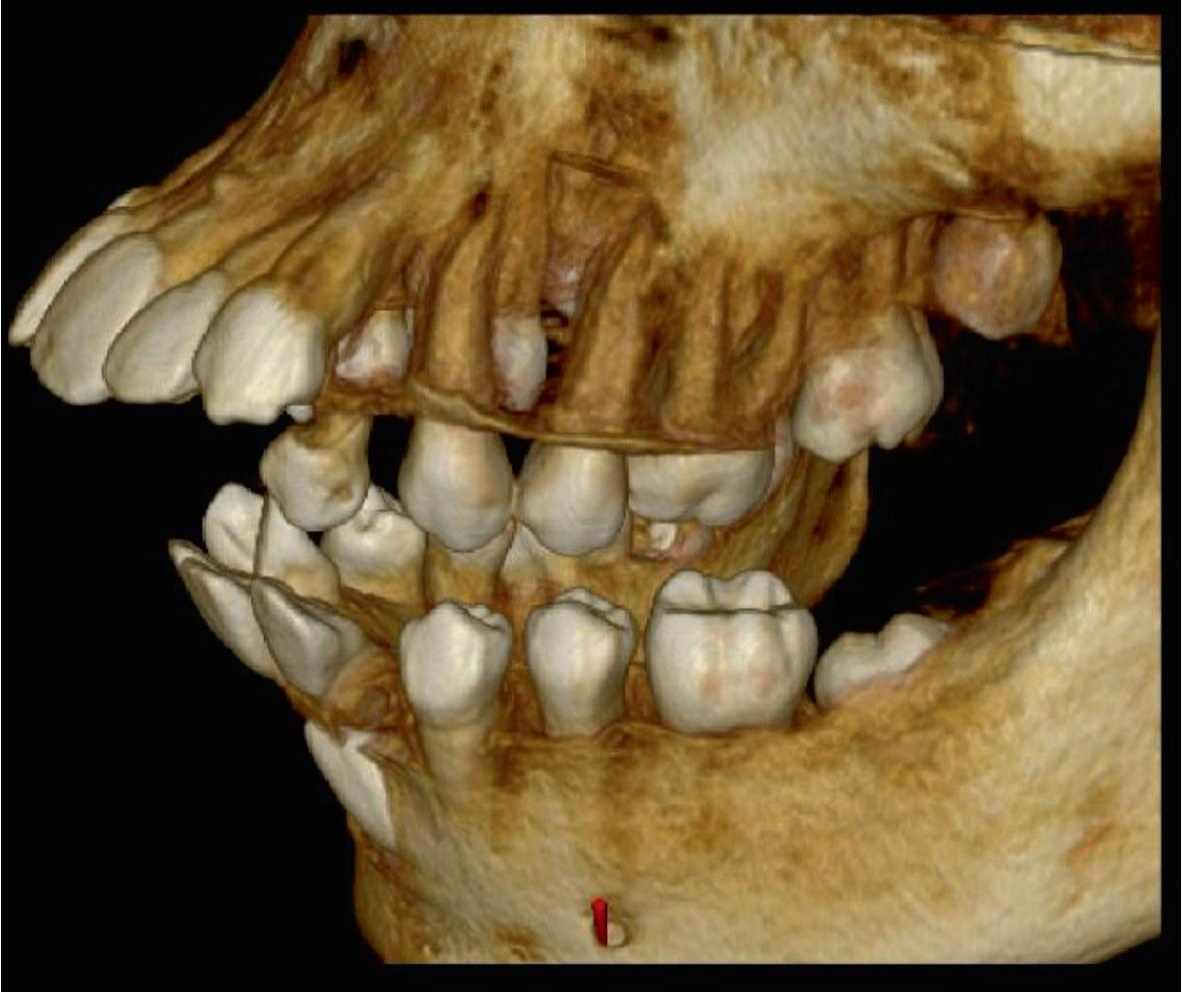
(d)

**Figure:**
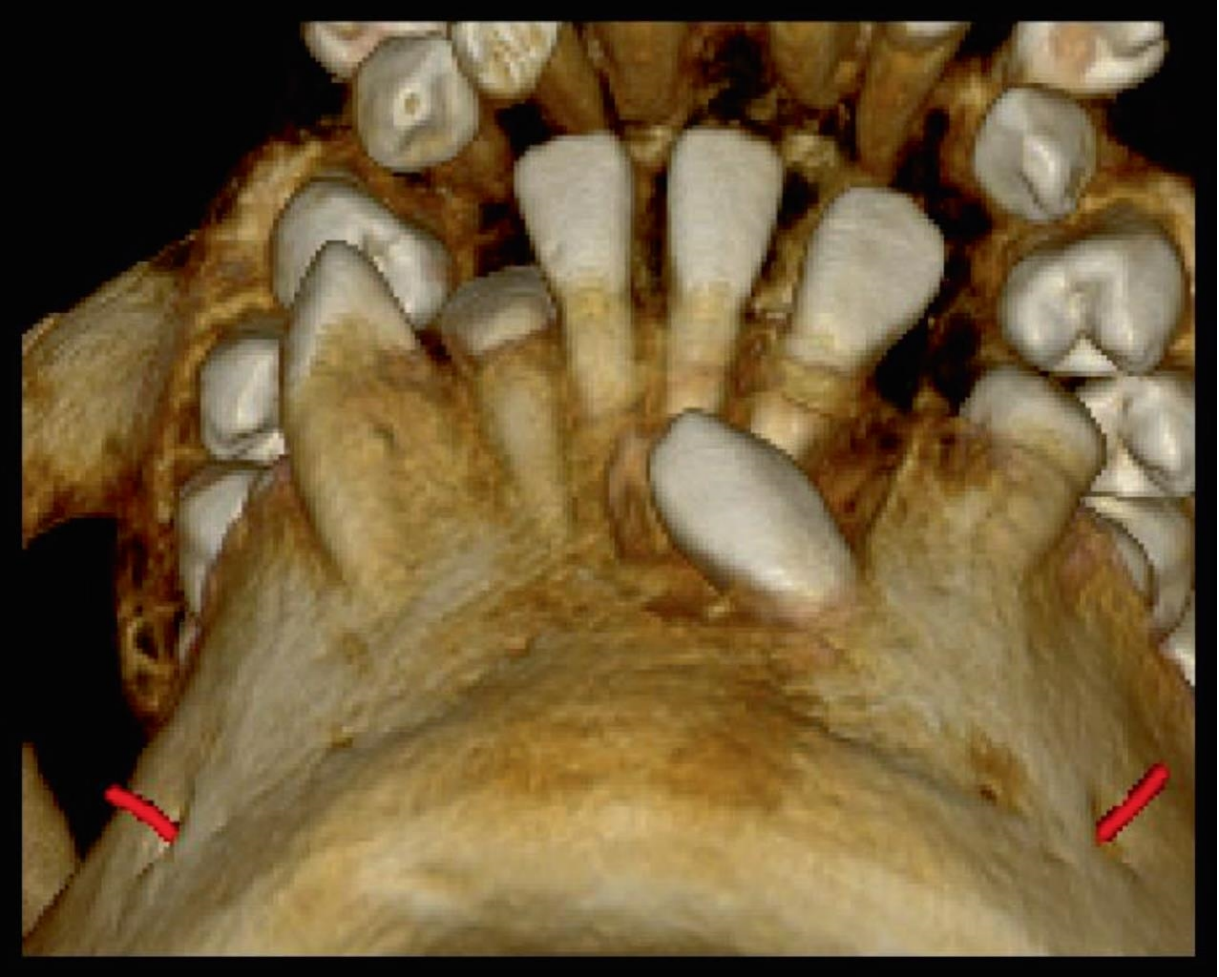
(e)

**Figure:**
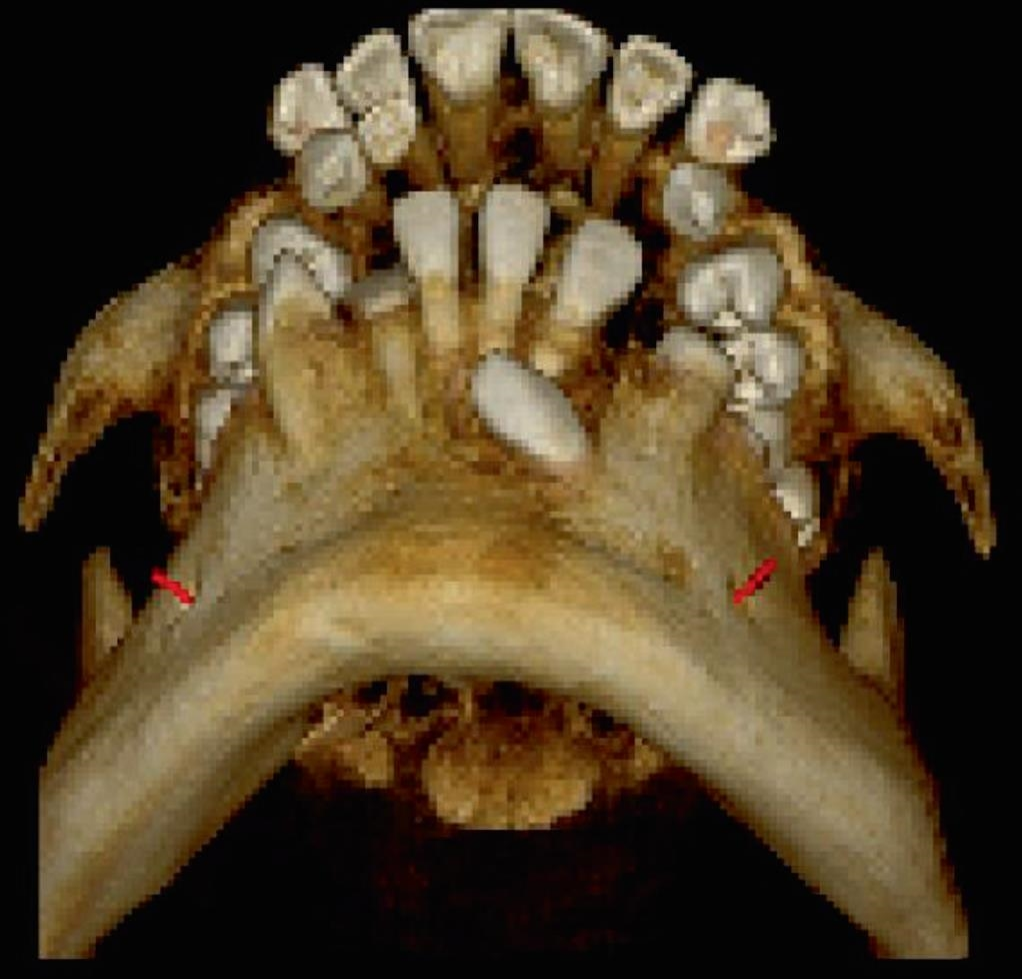
(f)

Initial dental treatment consisted of oral hygiene instructions, prophylaxis, and supragingival scaling. Primary teeth with high mobility were extracted. Periodontal evaluation showed that despite the gingival enlargement, probing depths were within normal limits (maximum 3 mm) with no bone loss, confirming a diagnosis of drug‐induced gingival enlargement rather than periodontitis.

Subsequently, pit and fissure sealing with ionomer cement was performed on all permanent first molar teeth. A ulectomy was performed on the permanent maxillary right canine and left lateral incisor to facilitate the eruption of these impacted teeth. All procedures were performed in the dental clinic and were well tolerated by the patient.

The main demands and future planning consist of periodontal treatment to improve gingival hyperplasia, followed by orthodontic treatment. However, to perform any invasive intervention, the patient′s general health condition requires the administration of general anesthesia and procedures performed in a hospital environment. Authorization was requested from the patient′s attending physician to perform the dental procedures that are still necessary.

## 3. Discussion

Unlike previous literature that focuses heavily on renal pathology, this report highlights the oral manifestations associated with Ask‐Upmark kidney. To our knowledge, this is the first case report describing the oral features of this specific congenital anomaly.

The most striking oral feature in this patient was severe gingival hyperplasia. This finding is consistent with drug‐induced gingival overgrowth, a known side effect of the patient′s chronic medication regimen, specifically amlodipine (a calcium channel blocker) and valproic acid (an anticonvulsant) [5, 6]. Drug‐induced gingival hyperplasia is a periodontal side effect of certain medications, causing swelling, bleeding, difficulties with chewing and speaking, as well as esthetic problems, which leads to a worsening of the patient′s quality of life [7, 8]. The pathogenesis involves alterations in gingival fibroblast metabolism and collagen degradation inhibition. Although a gingival biopsy was suggested, the diagnosis was established clinically based on medication history and the absence of periodontal pockets. Management focuses on oral hygiene instruction with meticulous self‐care, consultations for prophylaxis and gum scraping, and replacement of medication, which in many cases is sufficient to resolve gingival hyperplasia. In extensive cases, gingivectomy may be performed [6].

In addition to soft tissue changes, the patient exhibited significant dental anomalies, including delayed tooth eruption and impaction, which, along with systemic conditions, affect the patient′s quality of life [9–11]. In the present case report, unsatisfactory occlusion, gingival hyperplasia, and impaction of several teeth were observed, causing difficulty with performing oral hygiene, together with speech and eating problems. As the patient showed good behavior in the dental environment, initial interventions were performed to adapt the oral environment, and oral hygiene instruction was given to the patient and his guardians in order to maintain the results. Other demands will be met according to patient requirements and medical authorization with the aim of ensuring quality of life. In this context, constant communication between the dental and medical teams is outstanding to determine the ideal treatment.

Several systemic conditions demonstrate a clear association between oral abnormalities and kidney disease. For instance, enamel renal syndrome is an autosomal recessive disorder characterized by enamel hypoplasia, delayed or failed tooth eruption, intrapulpal calcifications, and gingival overgrowth [12–15]. Similarly, patients with chronic kidney disease may present with severe developmental enamel defects, including discoloration, pitting, and reduced hardness, which lead to extensive tooth wear even under normal functional loads. Furthermore, the alkaline oral pH resulting from uremia inhibits cariogenic bacteria, thereby reducing caries risk while increasing dental calculus accumulation [16–19]. Polycystic kidney disease is also known to cause dry mouth, oral ulcers, and infections [20]. In the present case, eruption disturbances may be linked to the systemic metabolic burden or localized bone density changes, despite the enamel structure appearing clinically normal.

The etiology of Ask‐Upmark kidney is not entirely understood, although it is believed that there is a relationship with renal vascular anomalies [3, 4]. The condition is associated with segmental infarcts in the cortical areas of the kidney, often attributed to stenosis or obliteration of renal segmental arteries [2, 21]. This compromised vascularization results in tissue ischemia, scarring, and cortical atrophy, leading to segmental nephropathy and, eventually, chronic renal failure, with nephrectomy being necessary in many cases [3]. In the present study, the patient′s kidney problems were diagnosed in childhood. Renal abnormality also has a strong relationship with vesicoureteral reflux, which can be observed in many patients [4, 22].

Diagnosis is challenging due to the segmental nature of renal lesions. Imaging exams showed areas of irregular cortical atrophy [3, 23, 24]. Magnetic resonance imaging and contrast‐enhanced computed tomography provided greater anatomical detail and could help identify associated vascular changes [21]. However, definitive confirmation of the diagnosis usually requires a renal biopsy, which reveals areas of interstitial fibrosis, tubular atrophy, and segmental glomerular sclerosis [4, 25]. As described in this case report, the family became aware of the child′s condition in the first years of life; from then on, follow‐up and adequate treatment were carried out, thus increasing the patient′s survival and improving their quality of life.

The prognosis is variable and depends on the extent of renal damage and the effectiveness of clinical management [3, 25]. In some cases, the disease can progress to terminal renal failure, whereas in others, renal impairment can remain stable for long periods, as reported in this study. At present, the patient has secondary chronic kidney disease with stable renal function and controlled systemic arterial hypertension. Regular medical follow‐up was essential to monitor disease progression and adjust treatment as necessary.

The treatment of Ask‐Upmark kidney is mainly conservative, focusing on controlling high blood pressure and preventing kidney complications through the use of antihypertensive agents or angiotensin receptor blockers [25]. Although advanced cases may require nephrectomy or renal replacement therapy [3, 25], the patient in this study has not required such interventions to date. Instead, the patient undergoes conservative treatment and regular follow‐up with a multidisciplinary team including nephrology, neurology, orthopedics, and physical therapy. Integral to this comprehensive care is the dental team, which plays a pivotal role in the patient′s quality of life. Consequently, early dental intervention and continuous communication with the medical specialists are paramount to manage potential complications safely.

## 4. Conclusions

Ask‐Upmark kidney is a rare condition that, while primarily renal, presents significant oral management challenges. This case illustrates the presence of severe drug‐induced gingival hyperplasia and tooth eruption disturbances. Knowledge of these oral manifestations allows for a more comprehensive, multidisciplinary treatment plan, improving the patient′s quality of life.

## Funding

This study was supported by Coordenação de Aperfeiçoamento de Pessoal de Nível Superior (10.13039/501100002322).

## Disclosure

The authors take full responsibility for the final content of the article.

## Consent

Consent was obtained from parent(s)/guardian(s).

## Conflicts of Interest

The authors declare no conflicts of interest.

## Data Availability

The data that support the findings of this study are available from the corresponding author upon reasonable request.
